# Effectiveness of IT-based interventions on self-management in adult kidney transplant recipients: a systematic review

**DOI:** 10.1186/s12911-020-01360-2

**Published:** 2021-01-02

**Authors:** Saeid Eslami, Farnaz Khoshrounejad, Reza Golmakani, Zhila Taherzadeh, Fariba Tohidinezhad, Sayyed Mostafa Mostafavi, Raheleh Ganjali

**Affiliations:** 1 Department of Medical Informatics, Faculty of Medicine, Mashhad University of Medical Sciences, Azadi Street, Mashhad, Iran; 2 Department of Medical Informatics, Academic Medical Center, University of Amsterdam, Amsterdam, The Netherlands; 3 Pharmaceutical Research Center, School of Pharmacy, Mashhad University of Medical Sciences, Mashhad, Iran; 4 Department of Emergency Medicine, Doctor Shariati Hospital, Mashhad University of Medical Sciences, Mashhad, Iran; 5 Neurogenic Inflammation Research Center, Mashhad University of Medical Sciences, Mashhad, Iran

**Keywords:** Kidney transplant, Information technology, Self-care, Systematic review

## Abstract

**Background:**

Kidney transplant outcomes are broadly associated with transplant recipients’ capacity in following a complex and continuous self-management regimen. Health information technology has the potential to empower patients. This systematic review aimed to determine the impacts of IT-based interventions for self-management in kidney transplant recipients.

**Methods:**

A comprehensive investigation was performed in MEDLINE (via PubMed) and EMBASE (via Scopus) in April 2019. Eligible studies were the randomized controlled trials which aimed to design an automated IT-based intervention. All English papers including adult kidney transplant recipients were included. To assess the clinical trial’s quality, Cochrane Collaboration’s assessment tool was employed. The articles were integrated based on category of outcomes, characteristics of interventions, and their impact. The interventions were classified based on the used IT-based tools, including smart phones, coverage tools, computer systems, and a combination of several tools. The impact of interventions was defined as: (1) positive effect (i.e. statistically significant), and (2) no effect (i.e. not statistically significant).

**Results:**

A total of 2392 articles were retrieved and eight publications were included for full-text analysis. Interventions include those involving the use of computerized systems (3 studies), smart phone application (3 studies), and multiple components (2 studies). The studies evaluated 30 outcomes in total, including 24 care process and 6 clinical outcomes. In 18 (80%) out of 30 outcomes, interventions had a statistically significant positive effect, 66% in process and 33% in clinical outcomes.

**Conclusions:**

IT-based interventions (e.g. mobile health applications, wearable devices, and computer systems) can improve self-management in kidney transplant recipients (including clinical and care process outcomes). However, further evaluation studies are required to quantify the impact of IT-based self-management interventions on short- and long-term clinical outcomes as well as health care costs and patients' quality of life.

## Background

Across the globe, most end stage renal disease syndrome (ERDS) patients undergo kidney transplantation [1].Kidney transplantation is regarded as the most effective therapeutic approach for people with ERDS. Kidney transplantation returns patients to daily life, increases their quality of life, and reduces the risk of mortality in the final stage of the disease [1–5]. In case of a transplant rejection, mortality rates and health care costs increase [6]. Given the limited health care resources due to growing demand, it is essential to create effective, non-interventional methods to promote self-management and monitor transplant recipients in order to increase the chances of success [7].

Transplant outcomes are broadly associated with transplant recipients’ capacity in following a complex and continuous self-management regimen, thus minimizing the risks of transplant rejection and related diseases [8, 9]. The ability to manage the outcomes of a chronic disease is defined as self-care. Self-management is classified into three groups: focusing on the disease needs; activating resources and living with chronic disease; and finally, managing drugs, roles, and feelings [10]. For kidney transplant recipients, self-management tasks include accepting medications, monitoring signs and symptoms of transplant rejection, performing regular periodic visits by specialists, compatibility with changes in social roles and communications, managing feelings, and creating new perspectives in life [11–13]. There is a need for more effective strategies to empower patients for self-management [14, 15].

The advent of health information technology (IT) and its related instruments have shown potential advantages for patients to actively participate in their health monitoring as well as assistance for health care providers [16]. Information technology has created new ways for providing health care and training to patients. Such improvements provide a basis for redesigning health care processes based on the use and integrity of electronic communications at all levels. It has been previously shown that information technology can empower patients. This technology is transforming the patient’s role from an inactive care services recipient into an active role in which patients are aware and involved in decision-making processes and have the right to choose [17]. In fact, a large number of researchers and system designers who previously focused on designing information technology software for care providers have now moved towards designing patient-focused software solutions [18].

Majority of interventional studies have examined the use of IT in designing patient training and monitoring for self-management in chronic diseases, including kidney transplant recipients. Some of these studies were effective, while others were ineffective. These heterogeneous results make it necessary to conduct a systematic review aiming to abstract the results of published studies. Several systematic reviews have been published on self-management patients with chronic kidney disease [19–21] and there is even a systematic study conducted on IT-based interventions to measure the self-management indices among patients with chronic renal disorders [22].

Although IT-based interventions have the potential to inform patients and improve self-care, there is a need for a provider to intervene in non-automated IT-based interventions; however, this would entail a potential source of human error. IT-based interventions can reduce error, improve self-management, and increase patients’ awareness and knowledge. None of the automated IT-based systematic reviews had focused on kidney transplant recipient patients while patients’ self-management behaviors will help them to improve clinical and process outcomes both before and after transplantation. In fact, this need begins before the transplantation, and as long as they continue to live with the transplanted kidney, this basic need continues to be present. Therefore, this study was conducted to describe the main features of the IT-based interventions and summarize their effectiveness on self-management outcomes among kidney transplant recipients. The main questions to be addressed on this topic include: What IT-based interventions studies have been conducted on kidney transplant recipients? What are the main characteristics of these studies? Did these interventions result in positive improvement on self-management outcomes (clinical and process care) among kidney transplant recipients?

## Methods

We performed this review according to Cochrane Handbook for Systematic Reviews of Interventions [23] and reported it by the Preferred Reporting Items for Systematic Reviews and Meta-Analyzes (PRISMA) [24].

### Data source and research strategy

We conducted a comprehensive search on Scopus and Medline (through PubMed) from 1980 until April 2019. The search was done using an arrangement of keywords and mesh terms that focused on self-management, empowerment, and participation as well as kidney transplantation. The combinations of keywords and MeSH terms in search strategies have been listed in Table 1.

**Table1:** Keywords and MeSH terms in the search strategy

Kidney transplantation	Keywords	Kidney transplantation, renal transplantation, kidney grafting, kidney transplant
	MeSH terms	Kidney transplantation
Self-management	Keywords	Self-care, disease management, self-management, decision aids, patient participation, patient involvement, medication alert system, reminder systems, patient education, patient empowerment, patient activation, patient engagement, patient participation, patient education, reminder systems, mobile health, telehealth
	MeSH terms	Self-care, disease management, reminder systems, patient participation, patient education, decision support techniques, clinical decision support systems, telemedicine

### Eligibility criteria

The factors used to determine the inclusion criteria were as follows: population, intervention, comparator, outcomes, and study design (PICOS). An IT-based system is defined as an automated system, without direct human intervention. To be included in the present study, the retrieved articles had to meet the following inclusion criteria: (1) IT-based interventions with an automated function, (2) interventions involving some type of IT-based tools to enable self-management, including smart phones, tablets, and computers, (3) published studies in scientific journals, (4) publication dated in the 1980–2017 period, (5) English language, (6) studies performed as a randomized clinical trial, which entails a control group which represents the standard care, without an IT-based intervention, and (7) studies performed on kidney transplant recipients.

The following articles were excluded from the study: (1) studies involving direct human interventions (e.g. non-automatic phone calls, Short Message Service (SMS) and video representation systems).

### Data extraction

After article retrieval, in the first phase of selection the articles’ title and abstract was used as the basis to decide whether they fit the research question domain. In the second phase, selection was based on the inclusion criteria as described above, extracting studies that used IT-based solutions for self-management of kidney transplant patients. Following these two phases of article selection, the full text of the remaining articles was further investigated, and the remaining articles were categorized based on the type of IT-based tools.

Following variables were extracted from included articles: characteristics of study sample, performance variables, method description, type of intervention, duration of study, as well as defined outcomes and results. The same authors (RG, FK) independently reviewed full text of selected articles to be included for qualitative synthesis. Disagreements were resolved by discussion with the third reviewer (SE).

### Risk of bias

The Cochrane Collaboration’s evaluation tool was used to assess the risk of bias in clinical trials [25]. This tool has been designed to assess risk of bias in term of following domains: selection bias (i.e. random sequence generation and allocation concealment), reporting bias (selective outcomes reporting), performance bias (i.e. blinding of participants and personnel), attrition bias (incomplete outcomes data), detection bias (blinding of outcome assessor), and etc.

### Synthesis and analysis

We did not conduct a meta-analysis because of inconsistencies in measured interventions outcomes. The articles combination was based on the classification of: outcomes, the type of intervention, and its impact. The outcomes of the studies were categorized into two groups: clinical and care process outcomes. Clinical outcomes are used to quantify or describe the severity of the disease, such as blood pressure. Care process outcomes are associated with improving the quality of care and physician–patient interaction [26]. We used the Sign test to assess the effect of proposed intervention in either direction (e.g. positive or negative) for clinical and care process outcomes.

Based on the type of IT-based tools used in the interventions, we classified the studies as follows:

1. Mobile-based tools (i.e. educational contents which were delivered via smart phone applications or SMS).
2. Wearable devices (i.e. hardware devices which can be used for automatic recording of physiological changes, such as Holter monitor devices).
3. Computer systems, which enable the patient to record and transmit data and through the internet.
4. Multi-component tools, which is a combination of more than one tool from the above mentioned tools [27–29].

We used the technology performance framework to classify IT-based systems according to their function. This means classification of these systems based on whether they could:

- Inform: Delivery media (e.g. text, voice, photo, and video).
- Instruct: Offer instructions to the user
- Record: Capture data entered by the user
- Display: Show or output data entered by the user
- Guide: Deliver guidance based on user provided information
- Remind/Alert: Provide alerts and reminders for specific tasks or at specific times to the user
- Communicate: Provide communication path between the user/patient and health care providers [28–30]

The impact of interventions was defined as: (1) positive (i.e. statistically significant) and (2) no effect (i.e. not statistically significant).

## Results

### Study selection

**Fig. 1:**
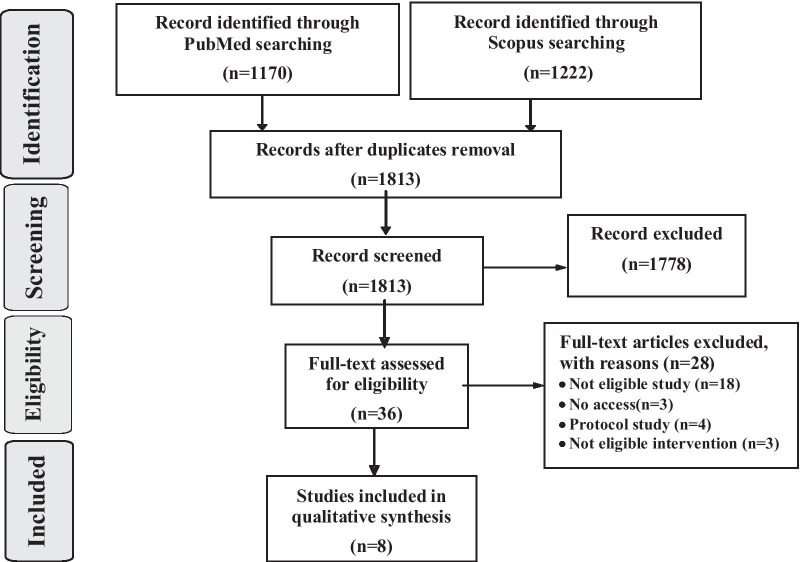
Flow diagram of the literature search and publication selection

Figure 1 (Additional file 1) demonstrates the study selection process. A total of 2392 records (1170 from PubMed and 1222 from Scopus) were retrieved. After deduplication, 1813 unique studies remained. After evaluation of titles and abstracts, based on the inclusion and exclusion criteria, 36 papers were selected for full-text evaluation, among which 28 papers were excluded, and 8 papers were selected to be included in the study. Table 1 shows the general characteristics of the selected studies. In full-text evaluation stage, 8 studies were selected, all published in English. The oldest study was published in 2010 [31], and the latest one was published in 2018 [32].

Out of these 8 studies, five studies were conducted in the United States [3, 33–36], two studies were conducted in Germany [22, 31] and one study was conducted in Canada [32]. Two studies included the patients on the waiting list of kidney transplant and six studies included kidney transplant recipients. The average sample size was 111 (21–288), and the median follow-up duration was 3 months (2 weeks–24 months). Majority of studies examined more than one (7/8 to 87.5%) outcome.

Out of these 8 studies, six studies were performed after transplantation. Other two studies examined three care process outcomes.

### Bias risk assessment

**Fig. 2:**
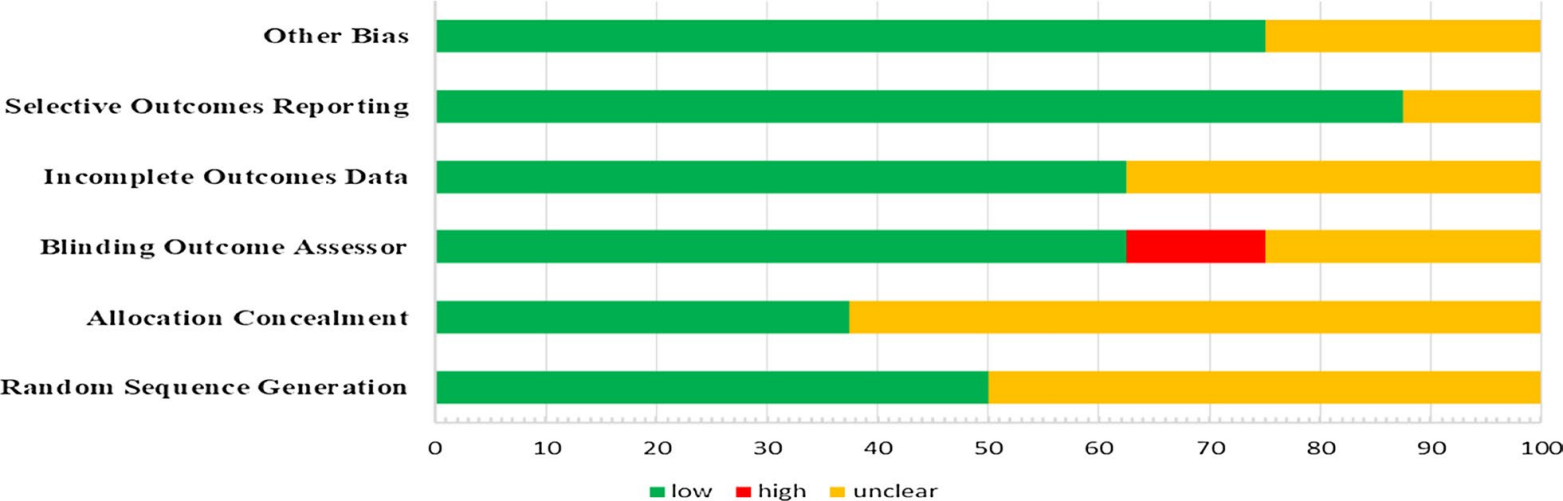
Risk of bias assessment of the included RCT studies

The quality evaluation results for the studies are shown in Fig. 2 (Additional file 2). About 50% of the studies clearly described the method used to generate the allocation sequence, and about 37.5% of the studies reported the allocation concealment. Less than 65% of the studies reported the method they used to deal with the incomplete data, and 50% of the studies provided insufficient information about the method they used to blind the outcome assessors. As observed in the studies, despite the presence of reporting protocol, the outcomes were reviewed based on the pre-specified and reported outcomes. This bias was low in 12.5% of the studies. The bias was also low in assessing the cause of participants' loss and exclusion from the study. There was incomplete data reporting in 37% of the studies. Totally, three studies had good quality [32–34] and one study had fair level of methodological quality [22].

### The impact of interventions on outcome measures

As shown in Table 2 a total of 30 outcomes variables were evaluated in the studies (i.e. 24 care process and 6 clinical outcomes). About 60% of the studies showed statistically significant positive effect in favor of using the proposed interventions. In the other 12 outcomes (40%), no significant difference was observed between intervention and control groups. Majority of outcomes were evaluated in interventions after transplantation (27/30, 90%).

**Table 2:** General characteristics of the included studies

Source, author, year, country	Participants	Sample Size (n)	Intervention	Duration	Outcomes	Results	Effect	Conclusion
Christina Freier et al., Germany [31]	Adolescents following transplantation	IG (26) CG (24)	Educational programme using the OTIS system (computer systems)	24 months	(1) Process outcome (IRK and IRB) (2) Clinical outcome (GFR)	(1) Overall IRK improved significantly over time (*p* < 0.0001) for IGr patients relative to CGr. Analysis of IRK demonstrated a significant increase in knowledge from T0 to T1 (*p* < 0.028) and from T1 to T2 (*p* < 0.045) in the IGr when compared to the CGr. With respect to IRB (2) The GFR gradient was stable in the IGr relative to a significant decrease in the CGr (*p* < 0.001)	(1) Positive effect (2) No effect	The results presented this medium holds the potential to improve perceived IRK and behavior. Moreover, this medium can support the challenging transition period from pediatric to adult care
Robinson et al., United States [36]	Kidney transplant recipient	IG (84) CG (86)	Educational tablet program (Smartphones/software)	2 weeks	(1) Process outcome (knowledge) (2) Process outcome (Recognize personal skin cancer risk) (3) Process outcome [Willingness to change sun protection (20–100 scale)] (4) Process outcome (sun-protection use) (5) Process outcome (Daily hours outdoors (0.5–6 h)	(1) The increase in knowledge of Hispanic Latino kidney transplant recipients was significantly greater than the increase in knowledge by non-Hispanic white and non-ispanic black kidney transplant recipients (*p* < .05) (2) Recognize personal skin cancer risk (1–5 scale) (3) The greatest willingness to change sun protection was demonstrated by Hispanic Latino kidney transplant recipients (*p* < .05) (4) use of sun protection increased from baseline to 2 weeks after the program in participants from all ethnic/racial groups in comparison with controls (*p* < 0.05) (4) Daily hours outdoors [0.5–6 h (*p* value = 0.01)]	(1) Positive effect (2) Positive effect (3) Positive effect (4) Positive effect (5) Positive effect	Kidney transplant recipients from diverse racial/ethnic groups and health literacy levels who used SunProtect became aware of their risk of developing skin cancer, increased their knowledge of skin cancer and sun protection, showed willingness to change their sun rotection, and changed their sun-protection behavior. Educational program with a tablet computer during the kidney transplant recipients’ 6- or 12-month follow-up visits to the transplant nephrologist improved sun protection in all racial/ethnic groups. Tablets may be used to provide patient education and reduce the physician’s burden of educating and training patients
McGillicuddy et al., United States [3]	Kidney transplant recipient	IG (11) CG (10)	mHealth system (include a BP monitoring device + electronic medication tray + a smart phone + text message) (Smartphones or PDA)	3 months	(1) Process outcome (medication adherence) (2) Clinical outcome (systolic blood pressure) (3) Clinical outcome (diastolic blood pressure)	(1) The repeated-measures ANOVA yielded a significant group by time interaction F 3,48 = 11.74, *p* < 0.001, and a significant main effect for time F 3,48 = 32.81, * p* < 0.001, 2) (2) A significant group by time interaction was observed for systolic BP (SBP), F 3,51 = 4.33, * p* = .009 (3) Results for diastolic blood pressure (DBP) also revealed a significant group by time interaction F 3,51 = 4.58, *p* = 0.006	(1) Positive effect (2) Positive effect (3) Positive effect	Prototype mHealth system was acceptable and resulted in significant improvements in medication adherence and BP control
Gordon et al., United States [33]	Hispanic kidney transplant candidates and their family Members and friends	IG (61) CG(62)	Website (computer systems)	3 weeks	Process outcome (knowledge about LDKT)	Website exposure was associated with a mean 21.7% same day knowledge score increase between pretest and posttest (*p* < 0.001). At 3 weeks, website participants' knowledge scores remained 22.6% above the pretest; control scores increased to 11.8% (*p* = 0.0001). Regression results found that website participants were associated with a 10.0% greater knowledge score at 3-week follow-up (*p* < 0.0001)	Positive effect	Our culturally targeted website increased participants' knowledge about LDKT (living donor kidney transplantations) above and beyond an in-person educational session in Spanish or English
Gordon, United States [34]	Kidney transplant candidate	IG(133) CG(155	Mobile Web application (Inform Me) (Smartphones/software)	3 weeks	(1) Process outcome (Knowledge about increased risk donor Kidney) (2) Process outcome (Willingness to accept increased risk donor kidney)	(1) Intervention participants had higher test 1 knowledge scores (mean difference, 6.61; 95% confidence interval [95% CI], 5.37–7.86) than control participants, representing a 44% higher score than control participants' scores. Intervention participants' knowledge scores increased with educational reinforcement (test 2) compared with control arm test 1 scores (mean difference, 9.50; 95% CI, 8.27–10.73). After 1 week, intervention participants' knowledge remained greater than controls' knowledge (mean difference, 3.63; 95% CI, 2.49–4.78) (test 3) (2) Willingness to accept an increased risk donor kidney did not differ between study arms at tests 1 and 3	(1) Positive effect (2) No effect	Inform Me use was associated with greater knowledge about increased risk donor kidneys above routine transplant education alone
Reese, United States [35]	Kidney transplant recipients	IG1(40) IG2(40) CG(40)	Wireless pill bottles monitoring with customized reminders (including alarms, texts, telephone calls, and/or e-mails) (multi-component system)	26 weeks	(1) Process outcome (bottle-measured tacrolimus adherence) (2) Clinical outcome (tacrolimus whole-blood concentrations)	(1) Mean adherence was 78%, 88%, and 55% in the reminders, reminders-plus-notification, and control arms (*p*, 0.001 for comparison of each intervention to control) (2) Mean tacrolimus levels were not significantly different between groups	(1) Positive effect (2) No effect	Provider notification and customized reminders appear promising in helping patients achieve better medication adherence, but these strategies require evaluation in trials powered to detect differences in clinical outcomes
Schmid, Germany [22]	Renal transplant recipients	IG (23) CG (23)	Remote telemonitoring And Real-time video consultations with access to significant medical data (multi-component system)	12 month	(1) Process outcome (Unplanned admission rate) (2) Process outcome (Length of unplanned stay) (3) Process outcome (Unplanned inpatient care costs) (4) Clinical outcome (acute Rejection rate) (5) Process outcome (rejection therapy initiation) (6) Clinical outcome [Estimated glomerular filtration rate (eGFR)] (7) Process outcome (Ambulatory care visit rate) (8) Process outcome (Immunosuppressive regimen Adherence) (9) Process outcome (Quality of life) (10) Process outcome (Return to employment)	(1) IG had significantly fewer unplanned admissions [IG (median = 0 admissions, interquartile range [IQR] = 1) versus CG (median = 2 admissions, IQR = 2)] (2) IG (median = 0 days, IQR = 6) versus CG (median = 13 days, IQR = 23) (3) The 23 RTR in the CG were hospitalized 29 times more often and spent 283 days longer in inpatient care, with correspondingly greater costs (4) The CG suffered two graft losses, the IG none (5) Biopsy-proven acute rejection rates were too low for permit comparative analyses (6) Both groups maintained transplant function: the median difference for the change in estimated GFR was + 3.6 mL in IG versus + 0.6 mL in CG (7) Differences in ambulatory care visit rates did not reach statistical significance (IG: median = 43 visits, IQR = 22. CG: median = 45 visits, IQR = 28) (8) The prevalence of nonadherence was 17.4% in the IG versus 56.5% in CG (*p* = 0.013) (9) The scores differed from the SOCG most significantly at T4 (IG [median = 0, IQR = 0.2] versus CG [median = 0.4, IQR = 0.6], *p* = 0.004, r = 0.42) (10) The IG returned to full employment soon after discharge, as indicated by their median working time percentages, which remained stable throughout year 1 post-transplant. The CG differed significantly between the baseline assessment (median = 50%, IQR = 100) and month 3 post-transplant (median = 0%, IQR = 50), Z = 2.694, *p* = 0.006, and did not return to full employment within the first post-transplant year	(1) Positive effect (2) Positive effect (3) Positive effect (4) No effect (5) No effect (6) No effect (7) No effect (8) Positive effect (9) Positive effect (10) Positive effect	This comparative effectiveness research has demonstrated the potential of telemedically supported case management to optimize routine evidence-based post-transplant aftercare and support its application at tertiary care hospitals. It provides a basis for a multicenter randomized trial to verify these outcomes in the medium and long term
Côté et al., Canada [32]	Renal transplant recipients	IG (35) CG (35)	Interactive Web-based sessions hosted by a virtual nurse(computer systems)	6 months	(1) Process outcome (Medication adherence) (2) Process outcome (Self-Efficacy) (3) Process outcome (skills) (4) Process outcome (medication side effects) (5) Process outcome (Self-Perceived General State of Health)	No statistically significant differences between the groups or over time (1) *p* value = 0.2 (2) *p* value = 0.37 (3) *p* value = 0.39 (4) *p* value = 0.44 (5) *p* value = 0.38	(1) No effect (2) No effect (3) No effect (4) No effect (5) No effect	This study showed that the Transplant-TAVIE, a Web-based tailored nursing intervention, is acceptable and could constitute an accessible adjunct in support of existing specialized services. However, given that this treatment is life long, it is important to deploy interventions adapted to the different phases of the medication management continuum in order to support these patients more effectively

*OTIS* organ transplantation information system, *IRK* illness-related knowledge, *IRB* illness-related behavior, *GFR* Glomerular Filtration Rate, *eGFR* estimated glomerular filtration rate, *BP* Blood Pressure, *mHealth* mobile health, *LDKT* living donor kidney transplant, *PDA* personal digital assistant

Out of 30 outcomes, 16 process of care and 4 clinical outcomes were reanalyzed using the Sign test. It should be noted that one study was excluded from the analysis due to incomplete baseline data [22]. The sign test was performed for clinical and process of care outcomes and showed that IT-based interventions were significantly affected the process of care outcomes (*p* = 0.021). However, no effect was observed for the clinical outcomes (*p* = 0.62). Seven studies [3, 31–36] evaluated sixteen process of care outcomes from which four studies had poor methodological quality [3, 31, 35, 36]. Three studies affected the outcome in favor of the intervention [32–34]. Four clinical outcomes were assessed in three study and all of them had poor methodological quality [3, 31, 35] (Table 3).

**Table 3:** Summary of measured effects of IT-based interventions

Outcome category	Outcome	Total	Effect		Effective interventions	Ineffective interventions
			Positive effect N (%)	No effect N (%)		
Clinical outcome (n = 6)	GFR	2		2(100%)		Computer systems (1) Multi-component systems(1)
	Systolic blood pressure	1	1(100)		Smartphones or PDA (1)	
	Diastolic blood pressure	1	1(100)		Smartphones or PDA (1)	
	Tacrolimus whole-blood level	1		1(100)		Multi-component systems(1)
	Acute Rejection rate	1		1(100)		Multi-component systems(1)
Process of care (n = 24)	IRB and IRK	1	1(100)		Computer systems(1)	
	Knowledge	3	3(100)		Smartphones or PDA (2) Computer systems (1)	
	Recognize personal skin cancer risk	1	1(100)		Smartphones or PDA (1)	
	Willingness to change sun protection	1	1(100)		Smartphones or PDA(1)	
	Sun-protection use	1	1(100)		Smartphones or PDA(1)	
	Daily hours outdoors	1	1(100)		Smartphones or PDA(1)	
	Medication adherence	4	3(75)	1(25)	Multi-component systems (2) Smartphones or PDA (1)	Computer systems(1)
	Willingness to accept increased risk donor kidney	1		1(100)		Smartphones or PDA(1)
	Unplanned admission rate	1	1(100)		Multi-component systems(1)	
	Length of unplanned stay	1	1(100)		Multi-component systems(1)	
	Unplanned inpatient care costs	1	1(100)		Multi-component systems(1)	
	Rejection therapy initiation	1		1(100)		Multi-component systems(1)
	Ambulatory care visit rate	1		1(100)		Multi-component systems(1)
	Quality of life	1	1(100)		Multi-component systems(1)	
	Return to employment	1	1(100)		Multi-component systems(1)	
	Self-Efficacy	1		1(100)		Computer systems(1)
	skills	1		1(100)		Computer systems(1)
	Medication side effects	1		1(100)		Computer systems(1)
	Self-perceived General State of Health	1		1(100)		Computer systems(1)
	Total	31	Clinical (2) Process (16) 18(60)	Clinical (4) Process (8) 12(40)	Computer systems (2) Multiple components (7) Smartphones or PDA(9)	Computer systems (6) Multiple components (5) Smartphones or PDA(1)

*GFR* glomerular filtration rate, *IRK* illness-related knowledge, *IRB* illness-related behavior

#### Clinical outcomes

The clinical outcomes evaluated in these studies were Glomerular Filtration Rate (GFR) changes (2 study), systolic blood pressure and diastolic blood pressure (1 study), tacrolimus whole-blood level (1 study) and acute rejection rate (1 study). Overall, the impact of IT-based interventions on clinical outcomes was significantly positive in 1 of the studies (33%), while in the other two studies (67%) there was no significant difference between the control and intervention groups.

In one study, the impact of computer-based patient education intervention on GFR changes was evaluated, having no significant effect [31]. In a study by McGillicuddy et al., the effect of an mHealth system (a blood pressure monitoring device) was evaluated on blood pressure, which also found a significant positive effect [3]. Another study evaluated the effect of IT-based interventions on the amount of tacrolimus, showing no significant difference between the intervention and control groups [35]. In another study, the effect of telemonitoring and real-time video consultations with access to significant medical data was evaluated on rejection rate and GFR, having no significant effect [22]. All clinical outcomes (6/6, 100%) were evaluated in interventions after transplantation.

#### Care process outcomes

The care process outcomes evaluated in this study consisted of patient’s knowledge (3 outcomes), IRB and IRK (1 outcome), recognition of personal skin cancer risk (1 outcome), willingness to change sun protection (1 outcome), sun-protection use (1 outcome), daily hours outdoors (1 outcome), medication adherence (4 outcome), willingness to accept increased risk donor kidney (1 outcome), unplanned admission rate (1 outcome), length of unplanned stay (1 outcome), unplanned inpatient care costs (1 outcome), rejection therapy initiation (1 outcome), ambulatory care visit rate (1 outcome), quality of life (1 outcome), return to employment (1 outcome), self-efficacy (1 outcome), skills (1 outcome), medication side effects (1 outcome), self-perceived general state of health (1 outcome).

All in all, the impact of IT-based interventions on care process outcomes was significantly positive in 16 of 24 (66%) outcomes. In three outcomes, the effect of intervention on medication adherence was significantly positive; in one study, the use of an m-Health system (BP monitoring device) and in the other study, the use of wireless pill bottles monitoring with customized reminders (including alarms, texts, telephone calls, and/or e-mails) were evaluated on tacrolimus adherence [3, 35]. In another study, the effect of tele-monitoring and real-time video consultations with access to significant medical data were evaluated on immunosuppressive adherence, which was found to have a positive effect [22]. On the other hand, one other study showed interactive web-based sessions have no significant effect on medication adherence [32]. Also, in 3 studies that evaluated the impact of tablet, mobile, and website accessibility on knowledge enhancement, there was a significant difference between the intervention and control groups [33–35]. Also, in another study, the effect of computer-based education on IRK and IRB was reported to be significantly positive [31]. Also, in one study, recognition of personal skin cancer risk, the desire to change sun protection and sun-screen use significantly increased using mobile app interventions, and daily hours outdoors significantly decreased using an intervention through the tablet [35]. Also, in one study, the desire to accept IRD kidney significantly increased using mobile app interventions [34]. In one study, an intervention by remote tele-monitoring and real-time video consultations with access to significant medical data improved unplanned admission rate, length of unplanned stay, unplanned inpatient care costs, quality of life, and return to employment [22].

Another study evaluated the effect of interactive web-based sessions hosted by a virtual nurse on self-efficacy, skills, medication side effects, and self-perceived general state of health and found no significant effects [32].

About 88% (21/24) of the care process outcomes were assessed in IT-based interventions after transplantation. About 67% (14/21) of the care process outcomes were significantly improved after implementing IT-based interventions. On the other hand, 33% (7/21) of the care process outcomes were not significantly different between the control and intervention groups.

Two studies were performed before transplantation. These studies 12% (3/24) evaluated care process outcomes. One study assessed the effect of an educational website on pre-transplant knowledge and found positive effect [33]. One study showed significant effect of a mobile-web application on pre-transplant knowledge. However, the willingness to accept the increased risk of donor kidney was not significantly improved [34].

### Interventions classification based on the type of technology and characteristics

Table 4 shows a summary of classification of interventions based on the type of technology. Three studies evaluated the effect of smart phone interventions using mobile health, mobile web applications, and a tablet program. The functions of smart phones consist of informing, communicating, and instructing. Smart phones had significantly positive effect on 9 out of 10 outcomes and showed no effect on only one outcome [3, 34, 36]. Three studies assessed the effect of computerized system interventions using a computer-based educational program and website. The functions of computerized systems involved instructing, informing, and communicating. These studies showed that interventions positively improved 2 out of 8 evaluated outcomes and was evaluated ineffective in six outcomes [31–33]. Also, two studies evaluated the impact of multi-component technologies, including a wearable tool, accompanied by SMS and telephone calls, as well as remote tele-monitoring and real-time video consultations with access to significant medical data. Their functions include recording, displaying, informing, instructing and communicating. The effect of using multi-component interventions was assessed as positive on seven outcomes, while it had no effect on 5 outcomes [22, 35].

**Table4:** Classification of the interventions according to technology type and features

References	Classification of consumer health informatics	Technology platform	Technology functionality	Technology description
Freier et al. [31]	Computer systems	Computer-based educational programme	Inform	A computer program was designed to arrange clinical information about pre-transplant, transplant operation, and post-transplant recommended care. The program also offered relevant medication taking behaviors based on each patient’s medication regimen. An interactive quiz was also obtained at the end of each education session to provide neutral feedback (correct or incorrect answer)
Robinson et al. [36]	Smartphones or PDA	Tablet program	Inform Communicate	A tablet-based program was implemented to offer interactive recommendations about sunscreen behaviors (e.g. skin cancer, appropriate methods for getting sun exposure, protective clothing, and etc.). Personal concerns of patients were also discussed by a physician during a live chatting session
McGillicuddy et al. [3]	Smartphones or PDA	A blood pressure monitoring device and electronic medication	Remind/Alert communicate	A smartphone connected to a wireless blood pressure monitoring device was used to record encrypted physiological parameters and also text messages reminders were sent to assist regular blood pressure monitoring process
Gordon et al. [33]	Computer systems	Website	Inform Instruct communicate	A website was designed to present 5–10 interactive messages on each of the following items: risks of donation and its relevant immigrant, financial, and cultural issues as well as available treatment options. A link to detailed description of each subject was also provided for interested patients. Moreover, interactive multimedia contents (e.g. video, telenovela, photograph, quizzes, and games) were also offered
Gordon et al. [34]	Smartphones or PDA	Mobile application	Inform Instruct	The iPad app, Inform Me used Computer Adaptive Learning (CAL) method to personalize educational materials and content according to each KTC comprehension levels in 5 interactive chapters: Introduction, Definition of Increased Risk, Risks and Benefits, Screening for Infection, and Treatment and Follow-Up. The Introduction provides an orientation and instructions; the other 4 chapters educate and assess comprehension. Inform Me shows videos, animations, and graphics to depict complex concepts
Reese et al. [35]	Multiple component	Monitoring with customized reminders	Remind/Alert Record	An electronic adherence monitoring system including following features was provided to kidney transplant recipients: notifications on wireless pill bottle, phone calls playing recorded messages, and short text message or email reminders
A. Schmid et al. [22]	Multiple component	Telemonitoring real-time video consultations	Record Display Remind/Alert Inform Instruct communicate	A telemonitoring system containing following features was implemented: (1) standard quiz including multiple-choice questions were obtained once a day, (2) self-measured data were transferred through a secure web-based connection, (3) clinical recommendations were provided via voice mailbox, phone calls, and short text messaging, (4) continuous access to a physician was provided to discuss possible daily concerns of the patients, (5) remote case management by resident nephrologists which was triggered in case of acute disorders
José Côté et al. [32]	Computer systems (websites)	Transplant-TAVIE	Inform Instruct	Three interactive web-based sessions were design to improve patient’s self-efficacy by teaching medication intake behaviors as well as verbal persuasion. The web-site was built using 89 videos and animations, 58 PDF files, and 93 pages of educational content
SUM	Computer systems (3studies) Multiple component (2 studies) Smartphones or PDA (3studies)			

*OTIS* organ transplantation information system, *IRK* illness-related knowledge, *mHealth* mobile health, *cal* computer adaptive learning, *KTC* kidney transplant candidate, *STP* senior transplant physician, *TNCM* transplant nurse case manager, *UMC* University Medical Center, *RTR* renal transplant recipient, *Transplant-TAVIE* treatment, virtual nursing assistance, and education

## Discussion

This systematic review abstracted the clinical trials which evaluated the effect of IT-based interventions on the self-management outcomes among kidney transplant recipients. A total of 6 studies including 930 patients showed significant improvement on the self-management outcomes. Majority of studies reached statistically significant effects (about 50% on clinical outcomes and 88.8% on process outcomes). Majority of the IT-based interventions were performed after transplantation (75%). Following medias were used: smart phones, wearable devices, computer systems, and multi-component interventions. The positive effect of IT-based interventions on clinical outcomes among transplant recipients is in accordance with previous systematic reviews which included the IT-based interventions [37–40]. Therefore, it can be concluded that IT-based tools are a suitable type of intervention to control clinical outcomes in kidney transplant recipients. The result of sign test for clinical and process of care outcomes showed that IT-based interventions significantly affected the process of care outcomes. There have been many trends in the use of IT-based technologies to educate patients and come up with better therapeutic options for various types of disease. Educating patients on disease and treatment is an effective way to increase awareness and self-management; however, knowledge is considered as one of the care process outcomes, which has been emphasized in most articles under study [31, 33, 34, 36]. In fact, knowledge is a key outcome for self-management in kidney transplant patients. Knowledge has the ability to empower patients by enhancing awareness [41]. In this study, the effect of IT-based interventions on kidney transplant recipients’ knowledge was reported as positive, consistent with results of other studies [42–45].

Increasing knowledge about the disease is a crucial aspect of a patient’s capability for drug management [43]. There is an established relationship between medication adherence and clinical outcomes, so that non-adherence to medication is closely linked with hospitalization and increased rate of mortality [44]. In this regard, IT-based systems have the potential to improve transplant knowledge and increase adherence to immunosuppressive medications, thus providing self-management improvement, which, in turn, leads to reduced transplant rejection and improved quality of life [46]. For instance, reminders can specifically be used to target and change unintentional forms of behavior in non-adherent patients taking medications, such as amnesia. Reminders can also be used to improve drug adherence in all age groups [47]. Other systematic reviews have also confirmed the positive impact of reminders on improving drug adherence [48]. While some studies have reported a positive impact for the use of IT-based interventions on medication adherence [49–51], other studies have reported the impact of IT-based interventions as ineffective [52].

In the present review, we investigated the effect of m-Health on care process outcomes and found it to be positive. Previously it has been reported that using m-health solutions with different forms of applicability provide tools that can improve clinical outcomes [53, 54]. It is an established fact that m-health can be used to improve quality, monitoring, and study of health-related data. For instance, the use of personalized learning tools requires a more active involvement of patients in the self-management process [55]. Previous studies have also shown that m-health solutions can improve the symptoms of the disease using active self-management interventions [54, 56].

Blood pressure is an important clinical biomarker in kidney transplant patients because of its associated complications. In our study, it was shown that using IT-based interventions, we can better monitor and control this clinical outcome. Other previous studies have also reported that remote monitoring systems are helpful in controlling hypertension [57–59].

Only one study was free from the risk of bias [32]. Limited information about allocation concealment, random sequence, incomplete outcomes data, and blinding outcome assessor were the top four that contributed to the low risk of bias score in included studies.

One of the strength points of the present study was our comprehensive search strategy that collected a large number of studies, thus reducing the prospects of dropping relevant articles. Because only randomized clinical trials were included in this study and other types of studies were excluded, there was a lower risk of bias and the quality of publications was thoroughly examined.

One of the limitations of this study was lack of accessibility to conference papers. Another limitation of this study was the presence of heterogeneity in reported outcomes, which made meta-analysis not feasible. Future studies should consider a larger sample size that can increase the generalizability of the study, which would, in turn, increase the effectiveness of the desired outcomes. Half of the studies were conducted over a short period of time (less than a month). The duration of studies should be in accordance with the defined outcomes. Moreover, the studies are needed to be improved in terms of reporting bias. In another words, randomized controlled trials (RCTs) are potentially associated with low risk of bias.

## Conclusions

IT-based interventions such as m-Health, wearable devices, and computer systems can improve self-management in kidney transplant recipients (including clinical and care process outcomes). It is suggested that these interventions begin before kidney transplantation and continue thereafter.

## Supplementary Information

**Additional file 1.** Search strategy.

**Additional file 2.** Risk of bias of individual RCT studies.

## Data Availability

All data generated or analyzed during this systematic review are included in this published article [and its supplementary information files].

## Abbreviations

ESRD: End stage renal disease
OTIS: Organ transplantation information system
IRK: Illness-related knowledge
IRB: Illness-related behavior
GFR: Glomerular filtration rate
BP: Blood pressure
eGFR: Estimated glomerular filtration rate
mHealth: Mobile health
LDKT: Living donor kidney transplant
PDA: Personal digital assistant
IRD: Increased risk donor
CAL: Computer adaptive learning
KTC: Kidney transplant candidate
TNCM: Transplant nurse case manager
STP: Senior transplant physician
UMC: University Medical Center
RTR: Renal transplant recipient
Transplant-TAVIE: Treatment, virtual nursing assistance, and education
