# Supplementary material for: Effectiveness of IT-based interventions on self-management in adult kidney transplant recipients: a systematic review
Source: BMC Med Inform Decis Mak. 2021 Jan 2;21:2. doi: 10.1186/s12911-020-01360-2 (PMC7778800; doi:10.1186/s12911-020-01360-2)
Supplement: Supplementary file 1 — Additional file 1. Search strategy. [file 12911_2020_1360_MOESM1_ESM.docx]

**Additional file 1.** **Search strategy**

1. **PubMed**

| #1 | Search ((("Patient Education as Topic"[Mesh]) OR ("Education, Patient"[Title/Abstract] OR "Patient Education"[Title/Abstract] OR "Education of Patients"[Title/Abstract]))) Filters: Publication date from 1980/01/01 to 2019/04/17 | 89759 |
| --- | --- | --- |
| #2 | Search ("Medication Alert System*"[Title/Abstract] OR "Alert System*, Medication"[Title/Abstract] OR "System*, Medication Alert"[Title/Abstract] OR (("Reminder Systems"[Mesh]) OR "Reminder System"[Title/Abstract] OR "System*, Reminder"[Title/Abstract]) Filters: Publication date from 1980/01/01 to 2019/04/17 | 3419 |
| #3 | Search ((((("Decision Support Systems, Clinical"[Mesh]) OR "Clinical Decision Support*" OR "Decision Support*, Clinical" OR "Support*, Clinical Decision"))) OR (((("Technique*, Decision Support"[Title/Abstract] OR "Decision Support Technic*"[Title/Abstract] OR "Decision Analyse*" OR "Decision Modeling"[Title/Abstract] OR "Clinical Prediction Rule*"[Title/Abstract] OR "Prediction Rule*, Clinical"[Title/Abstract] OR "Rule*, Clinical Prediction"[Title/Abstract] OR "Decision Analysis*") OR ("Decision Support Techniques"[Mesh])))))) Filters: Publication date from 1980/01/01 to 2019/04/17 | 141454 |
| #4 | Search (("Decision Aid*"[Title/Abstract] OR "Aid*, Decision"[Title/Abstract]) OR "Decision Support Techniques"[Mesh]) Filters: Publication date from 1980/01/01 to 2019/04/17 | 74560 |
| #5 | Search (((((("Participation, Patient"[Title/Abstract] OR "Patient Involvement"[Title/Abstract] OR "Involvement, Patient"[Title/Abstract] OR "Patient Empowerment"[Title/Abstract] OR "Empowerment, Patient"[Title/Abstract] OR "Patient Participation Rate"[Title/Abstract] OR "Patient Activation"[Title/Abstract] OR "Activation, Patient"[Title/Abstract] OR "Patient Engagement"[Title/Abstract] OR "Engagement, Patient"[Title/Abstract]))) OR "Patient Participation"[MeSH Terms])))) Filters: Publication date from 1980/01/01 to 2019/04/17 | 27645 |
| #6 | Search ("Self Care"[Title/Abstract] OR "Care, Self"[Title/Abstract] OR "Self-Care"[Title/Abstract] OR "Self-Management" OR "Self-Management"[Title/Abstract]) Filters: Publication date from 1980/01/01 to 2019/04/17 | 81381 |
| #7 | Search (((Disease Management[MeSH Terms]) OR "Disease Management"[Title/Abstract] OR "Disease Manage*"[Title/Abstract] OR "Manage*, Disease"[Title/Abstract])) Filters: Publication date from 1980/01/01 to 2019/04/17 | 74934 |
| #8 | search (("Telemedicine"[Mesh]) OR "Mobile Health"[Title/Abstract]) OR "Health, Mobile"[Title/Abstract]) OR "mHealth"[Title/Abstract]) OR "m-Health"[Title/Abstract]) OR "Telehealth"[Title/Abstract])) | 30176 |
| #9 | Search (((((kidney transplantation[MeSH Terms]) OR ("Renal Transplant*"[Title/Abstract] OR "Transplant*, Renal"[Title/Abstract] OR "Grafting, Kidney"[Title/Abstract] OR "Kidney Grafting"[Title/Abstract] OR "Transplant*, Kidney"[Title/Abstract] OR "Kidney Transplant*"[Title/Abstract]))) Filters: Publication date from 1980/01/01 to 2019/04/17 | 87118 |
| #10 | #8 #7 OR #6 OR #5 OR #4 OR #3 OR #2 OR #1 | 354471 |
| #11 | #9 AND #10 | 1191 |
| #11 | Filters: English | 1170 |

1. **Scopus**

| ( TITLE-ABS-KEY ( ( "Medication Alert System" OR "Medication Alert" ) ) OR TITLE-ABS-KEY ( "Reminder Systems" OR "Reminder" ) OR TITLE-ABS-KEY ( "Decision Support Systems, Clinical" OR "Clinical Decision Support" OR "Support, Clinical Decision" ) OR TITLE-ABS-KEY ( "Decision Support Techniques" OR "Technique, Decision Support" OR "Decision Support Technic" OR "Decision Analysis" OR "Decision Modeling" OR "Clinical Prediction Rule" OR "Prediction Rule, Clinical" OR "Rule, Clinical Prediction" OR "Decision Analysis" ) OR TITLE-ABS-KEY ( "Telemedicine" OR "Mobile Health" OR "mHealth" OR "Telehealth" ) OR TITLE-ABS-KEY ( "self-care" OR "self-management" OR "self-administrated" ) OR TITLE-ABS-KEY ( "Disease Management" OR "Management Disease" ) OR TITLE-ABS-KEY ( "Aid Decision" OR "Decision Aid" ) OR TITLE-ABS-KEY ( "Patient Participation" OR "Patient Involvement" OR "Involvement Patient" OR "Patient Empowerment" OR "Empowerment Patient" OR "Patient Participation" OR "Patient Activation" OR "Activation Patient" OR "Patient Engagement" ) OR TITLE-ABS-KEY ( "Patient Education as Topic" OR "Education,Patient" OR "Patient Education" OR "Education of Patients" ) AND TITLE-ABS-KEY ( "Kidney Transplantation" OR "Renal Transplantation" OR "Transplantations Renal" OR "Kidney Grafting" ) ) AND ORIG-LOAD-DATE AFT 20180922 AND ( EXCLUDE ( SUBJAREA , "CENG" ) OR EXCLUDE ( SUBJAREA , "AGRI" ) OR EXCLUDE ( SUBJAREA , "ARTS" ) OR EXCLUDE ( SUBJAREA , "VETE" ) OR EXCLUDE ( SUBJAREA , "CHEM" ) OR EXCLUDE ( SUBJAREA , "EART" ) OR EXCLUDE ( SUBJAREA , "ECON" ) OR EXCLUDE ( SUBJAREA , "PHYS" ) ) AND ( EXCLUDE ( DOCTYPE , "cp" ) OR EXCLUDE ( DOCTYPE , "ed" ) OR EXCLUDE ( DOCTYPE , "no" ) OR EXCLUDE ( DOCTYPE , "le" ) OR EXCLUDE ( DOCTYPE , "ch" ) ) AND ( EXCLUDE ( LANGUAGE , "French" ) OR EXCLUDE ( LANGUAGE , "German" ) OR EXCLUDE ( LANGUAGE , "Spanish" ) OR EXCLUDE ( LANGUAGE , "Chinese" ) OR EXCLUDE ( LANGUAGE , "Italian" ) OR EXCLUDE ( LANGUAGE , "Japanese" ) OR EXCLUDE ( LANGUAGE , "Portuguese" ) OR EXCLUDE ( LANGUAGE , "Dutch" ) OR EXCLUDE ( LANGUAGE , "Croatian" ) OR EXCLUDE ( LANGUAGE , "Polish" ) OR EXCLUDE ( LANGUAGE , "Turkish" ) OR EXCLUDE ( LANGUAGE , "Czech" ) OR EXCLUDE ( LANGUAGE , "Danish" ) OR EXCLUDE ( LANGUAGE , "Korean" ) OR EXCLUDE ( LANGUAGE , "Norwegian" ) OR EXCLUDE ( LANGUAGE , "Serbian" ) OR EXCLUDE ( LANGUAGE , "Slovak" ) OR EXCLUDE ( LANGUAGE , "Swedish" ) ) | 1222 |
| --- | --- |
