# Supplementary material for: Effectiveness of IT-based interventions on self-management in adult kidney transplant recipients: a systematic review
Source: BMC Med Inform Decis Mak. 2021 Jan 2;21:2. doi: 10.1186/s12911-020-01360-2 (PMC7778800; doi:10.1186/s12911-020-01360-2)
Supplement: Supplementary file 2 — Additional file 2. Risk of bias of individual RCT studies. [file 12911_2020_1360_MOESM2_ESM.docx]

**Additional file2.**

**Table S1. Risk of bias of individual RCT studies.**

**Cochrane Collaboration's evaluation tool was used to assess the quality of clinical trials**

|  | Random Sequence Generation | Allocation Concealment | Blinding Participants And Personnel | Blinding Outcome Assessor | Incomplete Outcomes Data | Selective Outcomes Reporting | Other Bias | Quality |
| --- | --- | --- | --- | --- | --- | --- | --- | --- |
| Christina Freier et al.,2010,Germany[26] | Unclear | Unclear | Not Relevant | Unclear | Unclear | Unclear | Low | Poor |
| June K Robinson et al.,2015,United States[30] | Unclear | Unclear | Not Relevant | Low | Unclear | Low | Low | Poor |
| McGillicuddy et al.,2013,United States  [3] | Unclear | Unclear | Not Relevant | Low | Unclear | Low | Low | Poor |
| Elisa J. Gordon et al.,2016, United States[28] | Low | Low | Not Relevant | Low | Low | Low | Unclear | Good |
| J. Gordon, 2017, united states[27] | Low | Low | Not Relevant | Low | Low | Low | Unclear | Good |
| Peter P. Reese,2016 united states[29] | Unclear | Unclear | Not Relevant | Unclear | Low | Low | Low | Poor |
| A. Schmid, 2017, Germany | Low | Unclear | Not Relevant | High | Low | Low | Low | Fair |
| José Côté et al.,2018,Canada | Low | Low | Not Relevant | Low | Low | Low | Low | Good |
